# Gender Differences in Potentially Inappropriate Medication Use among Older Adults

**DOI:** 10.3390/ph16060869

**Published:** 2023-06-12

**Authors:** Monira Alwhaibi, Bander Balkhi

**Affiliations:** 1 Department of Clinical Pharmacy, College of Pharmacy, King Saud University, Riyadh 11149, Saudi Arabia; bbalkhi@ksu.edu.sa; 2 Medication Safety Research Chair, College of Pharmacy, King Saud University, Riyadh 11149, Saudi Arabia; 3 Pharmacoeconomics Research Unit, College of Pharmacy, King Saud University, Riyadh 11451, Saudi Arabia

**Keywords:** elderly, ageing, Beers criteria, inappropriate prescribing

## Abstract

Background: Use of potentially inappropriate medication (PIMs) is a prominent concern that leads to significant medication-related issues among older adults. Notably, older women tend to utilize more medicines than men; older women frequently take more drugs. In addition, some evidence suggests that prescription PIMs vary by gender. This study examines the gender-based variation in prescribing PIM among older adults in Saudi Arabia. Methods: A cross-sectional retrospective analysis of electronic medical records from a large hospital in Saudi Arabia was carried out. Patients over the age of 65 who received ambulatory treatment were included in the study. The utilization of PIM was assessed based on Beers criteria. Descriptive statistics and logistic regression were employed to describe patterns of PIM utilization and identify factors associated with their use. All statistical analyses were performed using Version 9.4 of the Statistical Analysis Software (SAS^®^ 9.4). Results: The study comprised 4062 older people (age 65) who visited ambulatory care clinics; the average age was (72.6 ± 6.2) years. The majority of the study sample was women (56.8%). Among older adults, 44.7% of older men and 58.3% of older women reported having PIMs that should be avoided, indicating a higher prevalence of PIMs among women compared to men. In terms of the PIM categories used, women had a much higher utilization rate of cardiovascular and gastrointestinal drugs than men. In men, the use of PIMs was frequently associated with hypertension, ischemic heart disease, asthma, osteoarthritis, and cancer, while in women PIM use was associated with age, dyslipidemia, chronic kidney disease, and osteoporosis. Conclusions: This study revealed sex differences in PIM prescribing among older adults; PIM use is more common among women. Sex differences exist in clinical and socioeconomic characteristics and factors related to using potentially inappropriate medications. This study revealed essential areas that could be targeted by further interventions to improve drug-prescribing practices among older adults at risk of PIM.

## 1. Introduction

The rational use of medications is necessary for ensuring that patients receive appropriate medications that align with their clinical conditions and individual needs. This is crucial when considering older adults’ unique challenges and complexities. As individuals age, they often experience multiple chronic conditions and utilize multiple medications to manage their health conditions effectively. For instance, a retrospective analysis of 4713 hospitalized patients with chronic kidney disease (CKD) aged ≥65 years reported that 21.9% were taking at least one inappropriate medication at the time of hospital admission, which was linked to multiple medication use and end-stage CKD [1]. Another study on patients with heart failure (HF) and chronic obstructive pulmonary disease (COPD) found a significant underuse of beta-blockers among older patients [2]. Moreover, the study identified cases of inappropriate prescribing of beta-blockers, including the use of contraindicated medications, affecting approximately 30% of patients with HF and COPD. Thus, inappropriate medication use poses a significant concern, given that older adults are more susceptible to adverse drug reactions and drug–drug interactions.

Recently, growing attention and consideration has been given to individual characteristics, including gender differences, in the context of medication use. Gender is a pivotal element in various aspects of healthcare, including the prevalence of chronic illness, healthcare utilization, medication utilization, adherence to medications, use of self-medication, and health outcomes [3,4,5,6]. In addition, gender differences are known to be driven by biological factors such as metabolism and hormones, which may impact the pharmacokinetics and pharmacodynamics of medications and health-related behavioral risk factors such as lifestyle and healthcare provider communication [7,8].

Gender disparities have been reported in medication-related utilization and appropriate use of medications, with women at higher risk of potentially inappropriate medications (PIMs) and polypharmacy [6,9,10,11,12,13,14]. PIMs are defined as “medications that should be avoided due to their risk which outweighs their benefit and when there are equally or more effective but lower risk alternatives are available” [15]. The use of PIMs by older patients has a detrimental effect on their health outcomes; it has been associated with cardiovascular adverse events [16,17], drug-related problems as well as increased healthcare use and expenses, which can place a significant financial burden on older adults and society [18,19,20,21,22,23,24].

Few studies have examined sex differences in PIM use among older adults [6,9,10,11,12,13,14]. For example, using a retrospective cohort study design, Morgan et al. evaluated PIM use in 660,679 older adults aged 65 and older. The authors found that women had 16% higher odds of receiving PIMs than men (adjusted odds ratio (AOR) = 1.16, 95% CI = 1.12–1.21). Another retrospective cohort study using administrative data for 965,756 patients reported that women were more likely than men to receive inappropriate medications [12]. However, no research has yet assessed sex differences in PIM use or the causes of the reported discrepancies in the Saudi population. 

Several factors can contribute to these sex disparities, such as variances in morbidity and medication utilization patterns by men or women. Therefore, providing evidence based on real-world data might be helpful for exploring gender differences in PIMs used to optimize the use of medicines and avoid negative consequences on patients and the healthcare system. For this purpose, electronic health records are a valued source of information for exploring the prevalence of PIMs and identifying factors contributing to gender differences. Therefore, this study aimed to assess gender differences in the prevalence of PIM use using real-world data of elderly patients admitted to a large tertiary hospital. The findings from this study have the potential to identify specific medication utilization patterns that may contribute to these disparities and ultimately guide interventions to improve medication practice, reduce the use of PIMs and enhance the overall quality of care among older adults.

## 2. Results

### 2.1. Characteristics of the Study Sample

A total of 4062 older adults (age ≥ 65 year) who visited ambulatory care clinics were included in this study (Table 1). The mean age was (72.6 ± 6.2) years. The majority of the study sample was women (56.8%).

### 2.2. Sex Differences in Characteristics

Characteristics of the study sample by sex are presented in Table 1. Women had a significantly higher percentage of hypertension, diabetes, dyslipidemia, asthma, osteoarthritis, osteoporosis, and depression than men. For example, hypertension was more prevalent among women than men (62.4% vs. 37.6%, *p*-value < 0.0001). In addition, women had significantly higher use of potentially inappropriate medication than men (58.3% vs. 41.7%, *p*-value = 0.052). Moreover, women used more polypharmacy than men (60.5% vs. 39.5%, *p*-value < 0.0001). In addition, there was a significantly higher mean number of medications among women compared to men (6.72 versus 5.97, *p*-value < 0.0001).

### 2.3. Sex Differences in PIM Use

The prevalence of PIMs to be avoided was (41.7%) among older adults, 44.7% among older men, and 58.3% among older women (Table 2). One PIM was used by about 37.3% of the study sample, two PIMs by 9.6%, and three PIMs or more by 0.3%. PIMs to be used with caution were significantly higher among women compared to men (64.2% versus 35.8%, *p*-value < 0.0001). Additionally, women used cardiovascular and endocrine drugs much more than men in the categories of PIMs used.

### 2.4. Sex Differences in Sample Characteristics Related to PIM Use

Bivariate analysis of PIM use among older men was significantly higher in those who had diabetes, ischemic heart disease, and anxiety compared to those without those conditions (Table 3). For example, the rate of PIM use among older men was higher in those with diabetes than those without diabetes (53.5% vs. 38.7%, *p*-value < 0.0001). Moreover, PIM use was higher among older male patients with polypharmacy (59.9% vs. 22.3%, *p*-value < 0.0001) than those without polypharmacy use. 

Bivariate analysis PIM use among older women was significantly higher in those who have hypertension, diabetes, ischemic heart disease, and anxiety compared to those without those conditions (Table 3). For example, the rate of PIM use among older women was higher in those with diabetes than those without diabetes (56.3% vs. 41.6%, *p*-value < 0.0001). In addition, PIM use was higher among older women with polypharmacy (58.7% vs. 22.0%, *p*-value < 0.0001) than those without polypharmacy use.

### 2.5. Sex Differences in Factors Affecting PIM Use from Adjusted Logistic Regressions

The adjusted odds ratios (AORs) and 95% confidence intervals (CIs) for factors associated with PIM use among men and women are displayed in Table 4. PIM use was more likely among old men with diabetes, ischemic heart disease, asthma, osteoporosis, cancer, and anxiety. For example, men diagnosed with cancer were three times more likely to have used PIMs than those without cancer (AOR = 3.304, 95% CI = [1.71–6.36], *p* value = 0.0003). In addition, older men with polypharmacy use were five times more likely to have used PIMs compared to those without polypharmacy (AOR = 5.253, 95% CI = [4.09–6.74], *p* value ≤ 0.0001).

For factors associated with PIM use among women, PIM use was more likely among older women with diabetes, chronic kidney disease, and anxiety. For example, women with anxiety diagnosis were two times more likely to have used PIMs compared to those without anxiety (AOR = 2.473, 95% CI = [1.60–3.82], *p* value ≤ 0.0001). In addition, older women with polypharmacy use were five times more likely to have used PIM compared to those without polypharmacy (AOR = 5.094, 95% CI = [3.88–6.68], *p* value ≤ 0.0001).

## 3. Discussion

The findings of this study indicated that around one third of older adults who visited ambulatory care clinics filled one or more potentially inappropriate prescriptions. In addition, the prevalence of receiving potentially inappropriate medications was higher among women than men. These results emphasize the influence of clinical and socioeconomic characteristics on the utilization of potentially inappropriate medications among older adults. Thus, it is crucial to consider these factors when assessing the appropriate prescribing practices for this vulnerable population. 

The prevalence of potentially inappropriate prescription medications among older adults aligns with the estimate reported in other countries, most of which fall between 19.8% to 98.2% [25]. In terms of the most common types of PIM category used, our findings are consistent with those of other studies in that the majority of the elderly patients in this study used potentially inappropriate cardiovascular medications, which were substantially more frequently taken by women than by men. Furthermore, the results of our analysis that women have higher use of a potentially inappropriate prescription is in line with some earlier research that reported a higher prevalence in women compared to men [6,9,10,11,12,13,14].

Some results concerning gender differences in clinical characteristics suggest that biological factors shape the risk of potentially inappropriate medication use. Biological influences include gender differences in the prevalence of chronic health conditions for which medications may be inappropriately prescribed. In this study, hypertension, diabetes, dyslipidemia, asthma, osteoarthritis, osteoporosis, and depression were more prevalent among women than men. Given that women are more likely than men to be aware of chronic illnesses and seek medical care, it is plausible that women have a larger prevalence of chronic illnesses [26,27]; in the literature, studies have shown that women generally exhibit higher multimorbidity and chronic diseases than men [28]. Due to this biological reason, women may have higher health services and medication use, which, in turn, increases the risk of potentially inappropriate use. In addition, there are gender disparities in how medications are utilized and prescribed. A cross-sectional analysis of the Swedish Prescribed Drug Register database for all prescriptions supplied to the Swedish population revealed that women were prescribed more drugs than men across all age categories [29]. These results further confirm that gender differences in polypharmacy and PIMs should serve as an alarm to change certain healthcare practices and try to eliminate such discrepancies. It is crucial to address and eliminate these discrepancies in order to ensure equitable and safe medication management for all individuals, regardless of their gender. Healthcare providers need to be mindful of these gender disparities and tailor their prescribing practices accordingly to ensure appropriate medication management. Future research should explore these factors in more detail to gain a comprehensive understanding of the complex interactions between biological, socio-cultural, and healthcare system factors in shaping medication use patterns among older adults.

In the current investigation, after controlling for several patient-level characteristics, the most important common predictors for PIM use among older men and women were diabetes, ischemic heart disease, anxiety, and polypharmacy. Comorbidities have been cited as contributing factors to using PIM in several published studies [30,31,32,33,34,35,36]. In addition to specific medical conditions, the present study identified a significant association between polypharmacy and higher rates of PIM use. The use of PIMs is not uncommon when taking many medications at once; it is more likely to develop as a patient takes more prescription drugs [37,38]. This finding is consistent with previous research that revealed a positive correlation between polypharmacy and inappropriate medication use among older adults, which indicates the requirement of careful monitoring and regular medication reviews to minimize the potential harms associated with inappropriate prescribing [39]. Thus, the findings of this study highlight the need for healthcare providers to implement appropriate prescribing practices, particularly when managing older patients with specific comorbidities and those on polypharmacy. Healthcare professionals should carefully evaluate medication use’s potential risks and benefits, considering the patient’s clinical condition and overall medication regimen, as older adults are more vulnerable to adverse drug reactions and drug–drug interactions. Several drug interactions have been reported in the literature, highlighting their potential impact on patient safety and treatment efficacy. For instance, interactions between statins and antidepressants have been identified as a concern [40]. Statins, commonly prescribed for managing cholesterol levels, can interact with certain antidepressant medications, potentially altering their effectiveness or causing adverse effects. Similarly, interactions between atypical antipsychotics and anti-infective agents have also been reported [41]. These interactions can have implications for both drug classes’ therapeutic efficacy and safety, necessitating careful monitoring and consideration when prescribing medications. Additionally, studies have drawn attention to the impact of gender and sex on the pharmacodynamics and pharmacokinetics of drugs. It has been suggested that gender differences may impact the efficacy and safety of medications and their pharmacokinetic properties. For instance, specific hypertension treatments may exhibit variations in response based on gender-specific factors [42]. Understanding these nuances can help healthcare professionals tailor medication regimens to individual patients, considering their particular characteristics and potential gender-related differences. Furthermore, the high incidence of adverse drug reactions in women emphasizes the necessity to consider sex and gender differences in drug response. For instance, a study reveals significant variations between male and female COPD patients regarding quality of life and the use of inhaled drugs [43]. Compared to male patients, female patients had more symptoms to report and were more likely to be administered triple therapy. To avoid drug interactions, it is crucial to consider these gender-related aspects when prescribing drugs.

The National Institute for Health and Care Excellence (NICE) guidelines on medicine optimization have underlined that not every polypharmacy implies detrimental healthcare for the elderly [44]. It distinguishes between appropriate and inappropriate polypharmacy, with inappropriate polypharmacy being defined as the inappropriate prescription of many medicines or the failure to realize the desired benefit of the medicines. The Geriatric Society emphasizes the potential risk and complications associated with polypharmacy and PIMs, aiming to reduce medication-related adverse effects in older adults. Other societies, such as the American College of Cardiology/American Heart Association Task Force on Clinical Practice Guidelines, advise better adherence to preventive measures to maintain health and well-being, often requiring multiple medications [45]. This paradox highlights the complexity of medication management and the importance of balancing medication use. Thus, personalized management approaches that tailor medication regimens to specific individual needs while considering the potential risks and benefits of multiple medications are essential for promoting older adults’ overall health and quality of life.

### 3.1. Practical Implications

This study provides essential clinically pertinent information to inform healthcare professionals about the sex differences for PIM in older patients in a practical outpatient setting. To ensure that important safety precautions are taken when treating older patients, the roles of healthcare professionals may be expanded. By identifying areas where sex disparities exist in PIM use, targeted strategies can be designed to reduce inappropriate prescribing and enhance medication appropriateness. Inappropriate prescription and usage of medications can also be prevented by adequately integrating pharmacy services, such as continuous medication review. The most effective intervention, according to a thorough analysis of 47 studies using 52–124,802 patients and a range of interventions, including medication reviews, instructional strategies, clinical decision support systems, and organizational and multidimensional methods, was medication review [46]. Implementing regular medication reviews can help identify and address potential issues related to PIMs in older adults.

Furthermore, PIM use imposes a significant financial burden, and thus the cost associated with PIMs should be considered when designing any potential intervention. Accordingly, addressing these issues and minimizing the cost of PIMs is crucial to improving health outcomes and alleviating the financial burden on individuals and society [47]. This can involve increasing awareness among older adults and their caregivers about the potential risks of inappropriate medication use, promoting medication safety and education initiatives, and advocating the inclusion of cost-effectiveness considerations in medication-prescribing guidelines.

Additionally, several standardized tools can be utilized in real-world settings in the assessment of PIMs in older persons; the Beers criterion list is one such tool to evaluate PIMs for older patients [48]. Another recommended screening technique for elderly persons is the STOPP/START criteria [49]; which offers a checklist approach to assess a patient’s medication and quickly identify potentially inappropriate prescriptions. Healthcare professionals should consider incorporating these tools in their practice to determine the appropriateness of medications prescribed and reduced rates of PIM use among older adults.

Furthermore, healthcare systems should focus on enhancing inter-professional collaborations and communication. Multidisciplinary teams of healthcare professionals such as physicians, pharmacists, and nurses can work together to conduct medication reviews, share expertise, and ensure comprehensive patient care. Education and training programs targeting healthcare professionals, especially those prescribing medications to older adults, should emphasize recognizing gender differences in medication use patterns and the associated risks. Increasing awareness of these disparities can help clinicians tailor their prescribing practices and implement appropriate interventions based on older patients’ individual needs and characteristics. Moreover, by considering gender differences in PIM, we can move towards a more personalized and patient-centered approach to medication management for older adults, ensuring that healthcare practices are optimized for both men and women [50].

### 3.2. Strengths and Limitations

This study has some limitations. First, using the electronic health record data, our measure of potentially inappropriate prescribing is based on pharmacy dispensing records; dispensing of prescribed drugs is not equivalent to the consumption of the medicines as some prescriptions will be written but not filled by patients; this may affect the estimate of potentially inappropriate prescribing. In addition, this study was cross-sectional; therefore, the causal relationship cannot be determined. Moreover, data were obtained from a single hospital which limited the generalizability and external validity of the study findings. However, using the EHR provides real-world data that enabled us to evaluate PIM use among a large sample of patients.

## 4. Methods

### 4.1. Design and Setting

Retrospective cross-sectional study design was used in this investigation, and patients older than 65 years of age. The study collected twelve-month data from the Electronic Health Records of a large tertiary hospital in Saudi Arabia. The Institutional Review Board (IRB) of King Saud University issued approval with number E-17-2580. Health records for patients were maintained on a password-protected computer with limited access and were encoded to safeguard patient confidentiality.

### 4.2. Measures

#### 4.2.1. Potentially Inappropriate Medications (PIMs)

The American Geriatric Society (AGS) 2019 Beers criterion was applied to classify PIM use into two categories: those that should be avoided and those that should be taken with caution [48]. The usage of PIMs was divided into two categories: (1) PIM users (i.e., those who use one or more PIMs) and (2) non-PIM users (i.e., those who do not use PIMs). The prevalence of PIM exposure was then separated into four levels based on the number of PIMs prescribed: 0 (reference, no PIM exposure), 1 (prescribed one PIM), 2 (two PIMs), and 3 or more (three PIMs or more).

#### 4.2.2. Other Variables

Independent variables included socio-demographics (age, gender, nationality, and marital status). Chronic health conditions were identified using the International Classifications of Diseases–9th edition, Clinical Modification (ICD-9-CM) diagnostic codes. Chronic conditions include hypertension, diabetes, dyslipidemia, heart failure, ischemic heart disease, chronic kidney disease, cancer, asthma and chronic obstructive pulmonary disease, arthritis and osteoporosis, depression, anxiety, and dementia. This study defined polypharmacy use as the concurrent daily use of five or more medicines. The literature typically uses this concept of polypharmacy use [51]. We computed the average number of medicines in each patient’s medical file using this criterion.

### 4.3. Statistical Analysis

Descriptive statistics were expressed as the mean and standard deviation (±SD) for continuous variables and frequencies and percentages for categorical variables. Bivariate analyses using Student’s *t*-test and Pearson’s chi-squared test were used to assess the difference in demographics and disease characteristics between patients with and without PIMs. A two-tailed probability value of 0.05 was regarded as statistically significant for all analyses. A logistic regression analysis was carried out to evaluate the relationships between PIM use and the patient’s age, gender, polypharmacy, and different chronic conditions. A 95% confidence interval (CI) and a significance threshold of 0.05 were used for all statistical tests. Data were analyzed using the Statistical Analysis Software version 9.4 (SAS^®^ 9.4).

## 5. Conclusions

Significant sex differences in older adults’ risk of receiving a potentially inappropriate prescription have been explained by the difference in clinical and socioeconomic factors that influence PIM use. This study’s findings suggest that women’s elevated risk of PIM use results from a high prevalence of chronic health conditions.

## Figures and Tables

**Table 1:** Characteristics of the Study Sample by Sex among Older Adults aged 65+ years (n = 4062).

	Total		Men		Women			
	N	%	N	%	N	%	*p* Value	Sig.
**Total**	4062	100	1755	43.2	2307	56.8		
**Age Mean (SD)**	72.62 (6.28)		72.74 (6.10)		72.53 (6.41)		0.273	
**# Rx Mean (SD)**	6.40 (3.29)		5.97 (3.31)		6.72 (3.24)		<0.0001	***
**# Conditions (SD)**	2.42 (1.21)		2.12 (1.18)		2.65 (1.18)		<0.0001	***
**Marital Status**							<0.0001	***
Single	157	4.3	47	29.9	110	70.1		
Married	3479	95.7	1655	47.6	1824	52.4		
**Nationality**							<0.0001	***
Saudi	3726	91.8	1568	42.1	2158	57.9		
Non-Saudi	331	8.2	184	55.6	147	44.4		
**Hypertension**							<0.0001	***
Yes	2999	73.8	1129	37.6	1870	62.4		
No	1063	26.2	626	58.9	437	41.1		
**Diabetes**							0.421	
Yes	2302	56.7	982	42.7	1320	57.3		
No	1760	43.3	773	43.9	987	56.1		
**Chronic Kidney Disease**							0.059	
Yes	118	2.9	61	51.7	57	48.3		
No	3944	97.1	1694	43.0	2250	57.0		
**Dyslipidemia**							<0.0001	***
Yes	2209	54.4	717	32.5	1492	67.5		
No	1853	45.6	1038	56.0	815	44.0		
**Ischemic Heart Disease**							<0.0001	***
Yes	253	6.2	157	62.1	96	37.9		
No	3809	93.8	1598	42.0	2211	58.0		
**Asthma**							<0.0001	***
Yes	400	9.8	119	29.8	281	70.3		
No	3662	90.2	1636	44.7	2026	55.3		
**Osteoarthritis**							0.595	
Yes	373	9.2	166	44.5	207	55.5		
No	3689	90.8	1589	43.1	2100	56.9		
**Osteoporosis**							<0.0001	***
Yes	343	8.4	19	5.5	324	94.5		
No	3719	91.6	1736	46.7	1983	53.3		
**Cancer**							0.014	*
Yes	120	3.0	65	54.2	55	45.8		
No	3942	97.0	1690	42.9	2252	57.1		
**Anxiety**							<0.0001	***
Yes	371	9.1	207	55.8	164	44.2		
No	3691	90.9	1548	41.9	2143	58.1		
**Depression**							0.196	
Yes	60	1.5	21	35.0	39	65.0		
No	4002	98.5	1734	43.3	2268	56.7		
**PIM Use**							0.052	
Yes	1978	48.7	824	41.7	1154	58.3		
No	2084	51.3	931	44.7	1153	55.3		
**Polypharmacy**							<0.0001	***
≥5	2912	71.7	1150	39.5	1762	60.5		
0 to 4 drugs	1150	28.3	605	52.6	545	47.4		

*t*-test was used to assess the association between age and number of medications and PIM use. N: Number; PIM: Potentially Inappropriate Medications; Rx: Medications; Sig.: Significance; #: Number. Asterisks (*) represent significant differences in PIM use; *** *p* < 0.001; * 0.01 ≤ *p* < 0.05.

**Table 2:** Potentially Inappropriate Medications for Older Adults aged 65+ years, according to the 2019 Beers criteria (n= 4062).

	Total		Men		Women			
	N	%	N	%	N	%	*p* Value	Sig.
**Average number of PIMs (SD)**	0.400 (0.62)		0.587 (0.72)		0.648 (0.75)		0.0102	*
**PIM Use to be avoided**								
Yes	1978	48.7	824	41.7	1154	58.3	0.052	
No	2084	51.3	931	44.7	1153	55.3		
**PIM Use to be used with caution**								
Yes	1518	37.4	544	35.8	974	64.2	<0.0001	***
No	2544	62.6	1211	47.6	1333	52.4		
**Number of PIMs to be avoided**								
No PIM	2084	51.3	931	44.7	1153	55.3	0.032	*
One PIM	1515	37.3	652	43.0	863	57.0		
Two PIM	391	9.6	145	37.1	246	62.9		
Three or more PIM	72	1.8	27	37.5	45	62.5		
**Number of PIMs to be used with caution**								
No PIM caution	2544	62.6	1211	47.6	1333	52.4	<0.0001	***
One PIM caution	1336	32.9	493	36.9	843	63.1		
Two PIM caution	170	4.2	46	27.1	124	72.9		
Three or more PIM caution	12	0.3	5	41.7	7	58.3		
**Most common Classification of PIMs prescribed**								
Cardiovascular	2367	58.3	817	34.5	1550	65.5	<0.0001	***
Gastrointestinal	1439	35.4	553	38.4	886	61.6	<0.0001	***
Endocrine	657	16.2	288	43.8	369	56.2	0.722	
Pain Medications (NSAIDs)	272	6.7	120	44.1	152	55.9	0.753	
Antidepressants	16	0.4	5	31.3	11	68.8	0.333	
Antipsychotics	8	0.2	5	62.5	3	37.5	0.270	
Antispasmodics	20	0.5	12	60.0	8	40.0	0.129	
Anti-infective	7	0.2	1	14.3	6	85.7	0.122	
Genitourinary	4	0.0	1	25.0	3	75.0	0.462	
Antiparkinsonian agents	2	0.0	1	50.0	1	50.0	0.846	

N: Number; NSAIDs: Nonsteroidal anti-inflammatory drugs; PIMs: Potentially Inappropriate Medications; Asterisks (*) represent significant differences in PIM use; *** *p* < 0.001; * 0.01 ≤ *p* < 0.05.

**Table 3:** Number and raw percent of PIM use by sex among older adults aged 65+ years (n = 4062).

	Men					Women				
	PIM Use		No PIM Use			PIM Use		No PIM Use		
	N	%	N	%	*p* Value	N	%	N	%	*p* Value
**Total**	824	41.7	931	44.7		1154	58.3	1153	55.3	
**Age Mean (SD)**	72.8 (6.05)		72.65 (6.15)		0.503	72.9 (6.44)		72.15 (6.3)		0.0046
**# Rx Mean (SD)**	7.50 (3.25)		4.62 (2.73		<0.0001	8.14 (3.14)		5.29 (2.65)		<0.0001
**# Conditions (SD)**	2.33 (1.23)		1.93 (1.11)		<0.0001	2.80 (1.18)		2.50 (1.17)		<0.0001
**Marital Status**					0.544					0.428
Single	24	51.1	23	48.9		50	45.5	60	54.6	
Married	771	46.6	884	53.4		900	49.3	924	50.7	
**Nationality**					0.486					0.81
Saudi	733	46.8	835	53.3		1079	50	1079	50	
Non-Saudi	91	49.5	93	50.5		75	51	72	49	
**Hypertension**					0.091					0.028
Yes	547	48.5	582	51.6		956	51.1	914	48.9	
No	277	44.3	349	55.8		198	45.3	239	54.7	
**Diabetes**					<0.0001					<0.0001
Yes	525	53.5	457	46.5		743	56.3	577	43.7	
No	299	38.7	474	61.3		411	41.6	576	58.4	
**Dyslipidemia**					0.1844					0.748
Yes	323	45.1	394	55		750	50.3	742	49.7	
No	501	48.3	537	51.7		404	49.6	411	50.4	
**Ischemic Heart Disease**					<0.0001					0.001
Yes	99	63.1	58	36.9		63	65.6	33	34.4	
No	725	45.4	873	54.6		1091	49.3	1120	50.7	
**Asthma**					0.082					0.661
Yes	65	54.6	54	45.4		144	51.3	137	48.8	
No	759	46.4	877	53.6		1010	49.9	1016	50.2	
**Osteoarthritis**					0.073					0.821
Yes	67	40.4	99	59.6		102	49.3	105	50.7	
No	757	47.6	832	52.4		1052	50.1	1048	49.9	
**Osteoporosis**					0.154					0.016
Yes	12	63.2	7	36.8		142	43.8	182	56.2	
No	812	46.8	924	53.2		1012	51	971	49	
**Anxiety**					<0.0001					<0.0001
Yes	127	61.4	80	38.7		120	73.2	44	26.8	
No	697	45	851	55		1034	48.3	1109	51.8	
**Depression**					0.95					0.626
Yes	10	47.6	11	52.4		18	46.2	21	53.9	
No	814	46.9	920	53.1		1136	50.1	1132	49.9	
**Polypharmacy**					<0.0001					<0.0001
≥5	689	59.9	461	40.1		1034	58.7	728	41.3	
0 to 4 drugs	135	22.3	470	77.7		120	22	425	78	

*t*-test was used to assess the association between age and number of medications and PIM use. N: Number; PIM: Potentially Inappropriate Medications; Rx: Medications; Sig.: Significance; #: Number.

**Table 4:** Adjusted Odds Ratios and 95% Confidence Intervals from Logistic Regression on PIM Use by Sex among older Adults aged 65+ years (n = 4062).

	Men			Women		
	PIM Use			PIM Use		
	AOR	95% CI	Sig.	AOR	95% CI	Sig.
**Age Mean**	0.991	[0.97–1.00]	0.3021	1.019	[1.03–1.035]	0.0181
**Marital Status**						
Single	1.494	[0.78–2.84]	0.2225	0.796	[0.52–1.20]	0.2769
Married (Ref.)						
**Nationality**						
Saudi	1.093	[0.77–1.55]	0.618	1.071	[0.72–1.58]	0.7325
Non-Saudi (Ref.)						
**Hypertension**						
Yes	0.762	[0.60–0.96]	0.0263	0.806	[0.61–1.06]	0.1233
No						
**Diabetes**						
Yes	1.794	[1.44–2.23]	<0.0001	1.563	[1.27–1.91]	<0.0001
No						
**Chronic Kidney Disease**						
Yes	0.946	[0.54–1.65]	0.8463	2.03	[1.01–4.06]	0.0454
No						
**Dyslipidemia**						
Yes	0.841	[0.67–1.05]	0.1268	0.797	[0.64–0.99]	0.040
No (Ref.)						
**Ischemic Heart Disease**						
Yes	1.602	[1.11–2.35]	0.0116	1.266	[0.76–2.09]	0.3582
No (Ref.)						
**Asthma**						
Yes	1.712	[1.12–2.61]	0.013	0.845	[0.63–1.12]	0.2527
No (Ref.)						
**Osteoarthritis**						
Yes	0.674	[0.47–0.96]	0.0305	1.085	[0.77–1.52]	0.6382
No (Ref.)						
**Osteoporosis**						
Yes	2.862	[1.01–8.18]	0.0498	0.727	[0.54–0.96]	0.0264
No (Ref.)						
**Cancer**						
Yes	3.304	[1.71–6.36]	0.0003	1.364	[0.66–2.80]	0.3986
No						
**Anxiety**						
Yes	1.501	[1.05–2.13]	0.0239	2.473	[1.60–3.82]	<0.0001
No (Ref.)						
**Depression**						
Yes	0.991	[0.39–2.49]	0.9842	0.591	[0.27–1.27]	0.1778
No (Ref.)						
**Polypharmacy**						
≥5	5.253	[4.09–6.74]	<0.0001	5.094	[3.88–6.68]	<0.0001
0 to 4 drugs (Ref.)						

AOR: Adjusted Odds Ratio; PIM: Potentially Inappropriate Medications; Ref.: Reference group; Sig.: Significance.

## Data Availability

Due to our IRB policy, the EHR dataset utilized during and/or analyzed during the current study is not publically available. However, the corresponding author can provide it upon reasonable request.
